# Do Consumers Care about Nutrition and Health Claims? Some Evidence from Italy

**DOI:** 10.3390/nu11112735

**Published:** 2019-11-11

**Authors:** Azzurra Annunziata, Angela Mariani

**Affiliations:** Department of Economic and Legal Studies, University of Naples Parthenope, 80133 Naples, Italy; mariani@uniparthenope.it

**Keywords:** nutritional claims, health claims, cluster analysis

## Abstract

This study investigates Italian consumer knowledge and use of nutrition and health claims (NHCs). Six specific claims are examined on the basis of a web survey carried out on a sample of 504 consumers. Our results show that there is little attention to NHCs and their use is not widespread; objective knowledge of the selected claims is fairly scant, generating misinterpretation and confusion about their real meaning. K-means cluster analysis allowed us to identify three segments of consumers, characterized by different levels in attention and use frequency of NHCs, with a specific profile in terms of motivation and nutritional knowledge. Our results suggest the advisability of policy interventions and communication efforts which target the three segments with a view to achieving greater attention to NHCs. In conclusion, to boost knowledge concerning the actual meaning of the claims and their relation with a healthy diet, especially to reach non-users, information should be provided both simply and clearly, avoiding the use of complex scientific terminology.

## 1. Introduction

Non-communicable diseases constitute a major cause of death, disease, and disability, and one in five deaths globally can be attributed to an unhealthy diet [1]. Among other measures to supply consumers with appropriate information, provision of nutrition information on packaged foods is an important instrument in promoting healthier eating habits [2]. Worldwide, three main formats for providing information can be identified: Nutrition facts panels, front-of-pack labels, and nutrition and health claims (NHCs). NHCs include any form of short text describing the specific nutritional contents or health benefits of a food product. NHCs have the potential to make consumers more aware in making choices, increasing understanding of specific nutrient–disease relationships, and are used by food manufacturers as marketing devices. In Europe, EC Regulation 1924/2006 reports the specific definitions for nutrition and health claims in order to help consumers make healthy and informed food choices, stimulate and protect innovation in the food market, and facilitate the circulation of foods bearing claims across EU member states [3].

For nutrition claims the Regulation stipulates that such claims are only permitted if they are listed in its annex and fulfil the conditions laid down in the Regulation itself. As regards health claims, the Regulation defines two main different types of claims: Those specified in Article 13.1 that pertain to ‘general function’ relating to the role of a nutrient or other substance in growth, development, and the functions of the body (13.1a), or psychological and behavioral functions (13.1b), and those specified in Article 14.1 that refer to disease risk reduction (14.1.a), or children’s development (14.1.b). The Regulation also specifies that the use of a nutrition and health claim is only permitted if the average consumer can be expected to understand the beneficial effects expressed in the claim. At the end of 2018, there were 261 authorized health claims in the EC (2018).

According to previous research, consumer demand for food products carrying NHCs has grown rapidly [4,5], but at the same time a high market failure rate of new products with this kind of claim is registered. In this regard, Lähteenmäki (2013) [6] suggests that claims can only lend added value if consumers understand the benefit(s) concerned and whether the benefit is relevant to themselves, suggesting the need for in-depth understanding of the main motivations underlying consumer preferences and the heterogeneity in their demand.

Recently the EU-funded FP project CLYMBOL examined a large number of HCs with reference to 10 EU countries (not including Italy), reaching interesting conclusions on the need for more targeted information and greater communication efforts to make claims easier to understand and effective in supporting informed choices by consumers [7,8,9]. Other research proposes a cross-country approach including Italy [10,11,12] while specific studies on Italian consumers are few and far between [13,14,15].

In this regard, this paper enriches the existing literature, providing results from an Italian representative sample testing the familiarity, credibility, and objective knowledge of specific NHCs. Italy represents an interesting case study which is worth investigating: Previous research showed that Italian consumers differed from their European counterparts in their attitudes towards products with NHCs, showing less familiarity with, and attraction towards, such products and little positive influence of NHCs on their healthiness perception [10,11,12].

The paper is structured as follows: Section 2 presents the research background; Section 3 illustrates the materials used in the study and the methodology; Section 4 reports the results; Section 5 contains a discussion of the main findings and implications, and the paper ends with some concluding remarks.

## 2. Research Background

The impact of NHCs on consumer preferences and purchase behavior has been extensively analyzed. As highlighted in a recent review, such studies have yielded somewhat divergent findings on the influence of both consumer characteristics and product-specific characteristics [16].

From a theoretical perspective, in accordance with the Motivation–Ability–Opportunity framework [17] consumers’ responses to NHCs are affected by the opportunity, i.e., the availability of NHCs on the market, the motivation to engage in processing the information and their ability to process the information, which is related to their nutritional knowledge and level of understanding of claims [7,18].

As for motivation, research has shown the following positive correlation: The more consumers feel the need for information about food, diet and health and, in particular, are interested in a healthy diet, the more likely they are to spend time searching for information [18,19]. Interest in healthy eating has been found to be a prominent driver in explaining consumer use of NHCs [7,12]. In accordance, the same correlation was found with reference to personal health or that of family and friends, as experiencing illnesses (included overweight problems) is a major motivation for seeking health-related information which has increased interest in NHCs [14,15,20,21].

As for the ability to process, previous research has shown that nutrition knowledge strongly influences food label use [22] and an understanding of nutrition information [23], while specifically for knowledge and NHCs the results are contradictory. Consumers with in-depth objective nutritional knowledge according to Ares and colleagues (2008) [24] evaluated food products with an NHC as healthier, and had a higher purchase intention; furthermore, lack of nutrition knowledge limits consumers’ abilities to understand or evaluate a health claim, thus leading to lower perceived credibility of such claims. Others found that higher levels of nutrition knowledge led to less trust in health claims [25]. Consumers with a low nutritional knowledge have been found in Italy to have greater interest in nutritional claims [14]; other studies found for health claims no correlation at all [26].

With reference to opportunity, in accordance with the existing literature, other factors closely linked to consumer responses to NHCs are familiarity, in the sense of previous exposure, and credibility [6,27], which in turn affects the importance and reported use of claims [12].

Other research has stressed the role of consumer knowledge and understanding of nutrition and health claims already in use. With regard to subjective understanding, Williams (2005) and Trijp and van der Lans (2007) [28,29], focusing on four EU countries, show that health claims are perceived by consumers to be somewhat difficult to understand. By contrast, focusing on objective understanding, i.e., whether consumer understanding is in accordance with the scientific dossier on the claim, some studies have provided evidence that people do not always understand health and nutrition claims as they are intended [28,30,31,32,33]. Recent research [34] confirms such evidence, finding that consumers tend to interpret health and nutrition claims differently from scientific experts and regulators.

Finally, as regards socio-demographic characteristics, it is widely agreed that older consumers and women are more interested in, have a greater preference for, or a greater intention to buy products labelled with NHCs [16]. The structure of the household and the presence of young children have also been found to be significant with respect to interest in health [25] and nutrition claims [15], while with reference to education and income, research provides contradicting indications. Some studies have found that less educated consumers and those with lower incomes are more interested in products with NHCs [15,35]; on the other hand, other studies refute any correlation with education [36].

Against this background our detailed research objectives were: (a) To analyze the degree of attention and use of NHCs by Italian consumers; (b) to analyze consumers’ evaluation and objective knowledge of specific NHCs; (c) to identify and evaluate interpersonal diversity, through the identification and profiling of consumer segments with different levels of nutrition and health claims interest, knowledge and use.

## 3. Materials and Methods

### 3.1. Questionnaire Content

In order to achieve the research objectives described above, a quantitative online survey was performed with a representative sample of the Italian population by age, gender and region. Based on the existing literature and in accordance with the Motivation–Ability–Opportunity framework, a structured questionnaire was designed and organized in different sections to measure the following aspects: (a) Motivation to pay attention to NHCs; (b) nutritional knowledge; (c) general attention and use of NHCs; (d) evaluation and knowledge of specific NHCs; (e) socio-demographics and health status (Table 1).

Motivations investigated in the first section concerned consumers’ general health interest in food choices and the need for health-related information. The former was assessed using four items adapted from the General Health Interest scale [37]. The latter was measured by means of three items proposed in previous studies on NHCs [7,8]. In both cases we used a five-point agreement Likert scale (1 = not at all–5 = completely agree). The reliability of the scale was verified with Cronbach’s α test. The items used and Cronbach’s α scores are reported in Table 2.

Respondents’ nutritional knowledge was assessed through the use of five multiple choice questions extracted from the nutrition knowledge questionnaire [38] in line with previous studies carried out on the same topic [14,15,35]. In particular, in accordance with [14,15], two questions aimed to assess consumers’ knowledge on general nutritional recommendations (i.e., the optimal number of fruit and vegetable portions to be consumed daily and the type of fats that must be reduced) and three concerned specific knowledge on carbohydrate, protein and fat content of food. The decision to select these five questions was due to their relevance to, and consistency with, the Mediterranean Diet model which is widely followed in Italy. In order to obtain a synthetic measure on nutritional knowledge a normalized index was constructed using the scores obtained by each question (0 if wrong, 1 if correct) ranging from 0 to 5.

Respondents’ attention and use of NHCs when shopping were detected by asking consumers how often they paid attention to NHCs on labels and their purchasing frequency of such products using a 5-point Likert scale (1 = never–5 = always) as in previous research [14,15]. Respondents were then asked to express their perceived ability to process NHCs using three items selected from previous research [7] (see Table 2). Also in this case the scale reliability was verified with Cronbach’s α test.

In the fourth section respondents had to evaluate six specific claims, respectively three NCs and three HCs selected from the European Commission register of authorized claims (reported in Table 3). The selection of claims was based on their presence on the Italian market [39] but also considered the diversity in nutrients and the difference in wording. In the case of NCs we deliberately chose claims related to so-called “less is more” products whose sales, according to a recent Nielsen survey, have increased considerably on the Italian market, with particular reference to no sugar added products, low in sodium, and energy-reduced products [40].

For HCs we chose both general function claims (Art. 13.1.a) and disease risk reduction (Art. 14.1.a). Claims related to children’s development and health (14.1b) were deliberately excluded in order to avoid the answers being affected by the different household composition of the sample. For each claim respondents expressed their degree of familiarity and credibility using a 5-point Likert Scale (1 = not at all–5 = very much), as proposed in previous research [7,8]. Subsequently, for each of the selected claims objective individual knowledge was assessed with multiple-choice questions. As for nutritional knowledge, two indices were computed, counting the number of correct answers for both health and nutrition claims. In addition, an index of objective claim knowledge was created (knowledge score ranging from 0 to 6).

The last section of the questionnaire included socio-demographic and health status-related variables that are considered important predictors of consumers’ use of nutritional and health information on labels [21,31]. Among socio-demographic data, gender, age, education level, occupation, family size, presence of children in the household and income were included. As for health status, in accordance with previous research, personal diet-related needs and the presence of health problems in households were investigated [8,12].

Questionnaire understanding and length were pre-tested with a pilot sample of 20 consumers before proceeding with the main survey.

### 3.2. Data Collection

Data were collected in June 2019 by a national market research company (Astra Ricerche) through computer-assisted web interviewing. A quota sampling method was applied. Respondents were selected based on age (limited to the range 18–70 years) and place of residence (based on the four Nielsen areas: Northwest, Northeast, Central, and South of Italy), in accordance with statistics reported by the Italian National Institute of Statistics (ISTAT) for the 2017 resident population [41]. The inclusion criterion used was that respondents should be responsible for their household food shopping.

The procedures for contacting participants and administering questionnaires were managed by the same market research company. Over 950 individuals were invited to participate in a survey by email. About 47% of individuals contacted did not accept the invitation or did not complete the questionnaire. The final sample consists of 504 respondents.

Data collection procedures were performed in accordance with the ethical standards protocol of the data collection company in full compliance with the 1964 Helsinki declaration and its later amendments. Participants gave their informed consent to participate in the study to the data collection company. All data were collected and processed anonymously, and each participant was associated with a specific temporary identifier code.

### 3.3. Data Analysis

Data analysis included descriptive statistics (frequency distributions), bivariate (i.e., chi-square test, *t*-test, one-way analysis of variance) and multivariate analysis. The internal consistency and reliability of the scales used were measured with Cronbach’s alpha coefficients. Normal distribution of the data was checked using a graphical test (histogram with normality curve), skewness and kurtosis indices.

K-means cluster analysis, performed using variables related to general attention to and use of NHCs, allowed the statistical units to be classified into a set of ‘exclusive and exhaustive’ clusters so as to maximize their internally homogeneous nature and externally heterogeneous nature and identify different consumer profiles.

Subsequently, in order to profile each cluster in terms of socio-demographic and attitudinal variables, cross-tabulation with Chi-square statistics and one-way ANOVA comparison of means with post-hoc Tukey tests were performed (Table 4; Table 5). All analyses were conducted with IBM SPSS Statistics 24.

## 4. Results

### 4.1. Socio-Demographic Characteristics and Health Status

Table 1 shows the socio-demographic characteristics of the survey respondents: 50.2% are female, the mean age is 45.38 years, and mean household size is three individuals. With reference to the level of education, 58% had a high school diploma while 15% had a bachelor’s degree. Almost 45% of respondents live in northern Italy; as regards employment, those employed are the largest category (21.3%), followed by pensioners (14%) and housewives (12.7%); 28% of respondents had children under 12 living at home and in 63.3% of cases respondents stated that they had an average annual income in line with the national average.

With regard to the variables related to special dietary needs, 33% of respondents stated that they were influenced in their food choices by health reasons. As regards personal and/or household members’ health status, 43% of the sample stated that they did not suffer from any of the pathologies, while high cholesterol (27%) and high blood pressure (26%) were the main personal or household pathologies.

### 4.2. Motivation and Ability to Process NHCs

With respect to general health interest in food choices (Table 2), respondents consider themselves particular about the healthiness of food (3.4) and consider it important to follow a diet rich in vitamins and minerals (3.5). In addition, with reference to the need for health-related information, respondents seem sensitive to being informed if one or more components of the foods chosen reduces a risk factor of developing a disease (3.6). In terms of perceived ability to process NHCs, respondents tend to consider themselves quite knowledgeable (3) and are quite confident about their ability to understand the claims (3.2).

### 4.3. Consumer Attention to, and Use and Evaluation of NHCs

With respect to the degree of attention and use of nutritional and health information on the label, 33% of respondents say they often pay attention to the nutritional panel while shopping. The level of attention is higher for NCs (40% often pay attention) but is lower in the case of HCs (29% often pay attention). Considering the level of NHC use, 36% of respondents state that they often buy products with an NC on the label, while this percentage decreases to 26% for HCs. Cross-tabulation shows that the high level of attention to nutritional panels is related to the high level of attention to, and use of, NHCs (*p*-value = 0.00).

As shown by Table 3, respondents generally stated a slightly higher level of familiarity with NCs than HCs. Pairwise comparisons *t*-test was performed in order to verify whether the differences between means are statistically significant. Among the NCs proposed the most familiar is “no sugar added” while among the HCs the most familiar is “Omega-3 fatty acids help to maintain a healthy cardiovascular system”.

With regard to the credibility level our results show that the level of credibility is generally lower than that of familiarity. Respondents are more confident towards HCs, among which Omega-3 fatty acids obtained on average the highest level of credibility.

### 4.4. Objective knowledge of NHCs

In terms of general nutritional knowledge, our results show that the average value of the sample’s nutritional knowledge index is quite low (2.37 mean value, S.D. 1.19). With reference to specific claims, many of the interviewees do not know the actual meaning of the selected claims. We constructed a normalized index using the scores obtained by each question used (assigning value 1 for a correct answer, otherwise value 0). The scores ranged from 0 to 6. The mean value of the ability to process index is quite low (2.7; S.D. 1.31).

**Figure 1:**
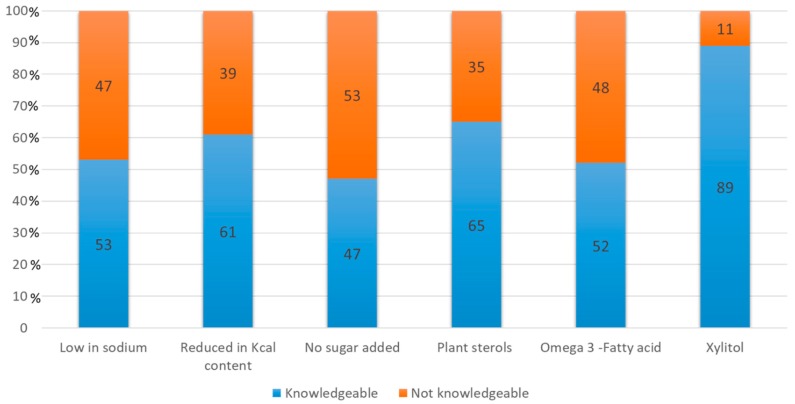
Share of interviewees who knew the actual meaning of the selected NHCs.

However, contrary to our expectations, failure to know the actual meaning is more marked for nutrition than for health claims (Figure 1). Among the nutrition claims, the claim that consumers stated was most familiar, namely “no sugar added”, proved the most difficult to interpret by consumers who were aware of the actual meaning of the claim, and in most cases considered that food reporting such claims contains no sugar at all or just contains less sugar (53%). Similarly but with less intensity “low in sodium” is misinterpreted by consumers, often indicating that products carrying this claim contain less sodium than other similar products or that the food was produced without sodium added (47%), while with reference to the claim “reduced in kcal content” the level of consumer knowledge is higher than the other two claims, with 61% of respondents indicating the correct answer.

For health claims respondents showed great difficulty in interpreting the claim “Omega-3 fatty acids help to maintain a healthy cardiovascular system”, which is the claim considered most familiar. Almost half the sample opted for the response that it helps to reduce the risk of heart attack or the level of cholesterol in the blood (48%). The degree of objective knowledge of the plant sterol claim is higher: 65% of respondents indicated the correct answer, while the meaning of the HC related to “xylitol in chewing gum” was the best known. This could be due to the fact that most chewing gum on the market is advertised in relation to this substance.

### 4.5. Consumer Segmentation

Applying K-means cluster analysis, three segments of consumers with different attention levels and use frequency of NHCs were identified. From the application of this method, the division into three segments was the optimal solution, where homogeneity is maximized within the individual clusters and minimized between them. In order to determine the optimal number of clusters the Calinski–Harabasz pseudo F-value was considered. The division into three clusters shows a higher value of the pseudo F’ test compared with other solutions tried. Differences between clusters in terms of attention and frequency of health and nutrition claims used are significant (*p* = 0.000).

The largest segment, namely “*Potential users*” in Cluster 1, accounted for 47% of respondents and included consumers who reported moderate levels of attention and use for both nutrition and health claims in line with the average sample.

Cluster 2, namely “*Claims users*”, accounted for 33% of respondents who reported the highest levels of attention and use of nutrition claims as well as health claims. Indeed, 35% of individuals in this cluster state they always pay attention to nutrition claims and 30% always use them. As for health claims 70% in this cluster state that they often pay attention to such claims and 68% often use them. The degree of attention towards nutritional panels is also higher in this cluster.

Cluster 3 accounted for 20% of respondents, showing the lowest levels of attention and use of nutrition claims and a very low level of attention and use of health claims. In this cluster, 49% rarely pay attention to nutrition claims and 20% never, which applies also to HC use. With regard to health claims, 41% never pay attention to them and 45% never use them while shopping. This cluster was therefore called “*Non-users*”.

The clusters were then profiled according to the degree of knowledge of specific claims and attitudinal determinants towards health and nutrition claims (Table 4) and socio–demographic characteristics (Table 5).

With respect to attitudinal determinants towards health and nutrition claims, Table 4 shows that the *Non-users* Cluster shows the lowest level of motivation to use NHCs, as confirmed by low levels of both general health interest in food choices and the need for health-related information. By contrast, the level of general health interest and need for health-related information is higher among *Claims users*, the cluster with the highest interest and use of nutrition and health claims. *Claims users* also consider themselves better able to interpret such claims.

With reference to specific claims, significant differences among clusters are revealed in terms of familiarity and credibility. Once again, *Claims users* includes consumers with the highest level of familiarity and credibility for both health and nutrition claims, and *Non-users* includes consumers with a lower degree of familiarity and credibility towards claims. Interestingly, for all three clusters HCs enjoy a higher degree of credibility than NCs.

As regards nutritional knowledge, consumers in the *Claims users* Cluster are more knowledgeable than consumers in the *Non-users* Cluster. Although the same result emerges for the NHC knowledge index, for the specific index for NCs actual knowledge is scant in all three clusters and no significant differences are noted. By contrast, as regards HC knowledge, consumers in the *Non-users* Cluster show a lower level compared to the other two clusters. No significant difference was detected in relation to objective knowledge of each specific claim used in the study.

With respect to socio-demographics, the *Non-users* cluster differs from the other two in the higher incidence of males with a lower level of education. Age does not differ significantly among groups, nor does the presence of children in the household, occupation, household economic status or area of residence. The incidence of individuals with personal and/or household members’ health problems that influence food choices is significantly different among clusters. The *Claims user* cluster reports the highest incidence of individuals with personal and/or household members’ health problems, which suggests that the presence of personal or family pathologies generates a greater need for information and greater attention to claims in this cluster.

## 5. Discussion

This paper explores the interest and use of NHCs in Italy, providing results from a national representative sample. Overall our results showed that attention to, and use of, NHCs is not very widespread among Italian consumers. At the same time, although consumers consider themselves quite capable of understanding claims, when objective knowledge is detected, the level of understanding of the selected claims is quite low, with misinterpretation and confusion being generated about the real meaning of the claims for both nutrition and health.

The degree of attention and use is higher for NCs than for HCs, both on average and for the six specific claims used in our study. However, the degree of familiarity as well as credibility of NHCs varies according to the claim considered, confirming that consumers’ responses to NHCs are strictly connected to the specific claim as reported elsewhere [15,29]. As for familiarity, among NCs consumers show the most familiarity with the “No sugar added” claim. This could be due to the fact that in recent years Italian consumers have shown particular sensitivity towards sugar-free products, which also represent a particularly growing market trend at this time [39,40]. Among HCs, consumers show that they have great familiarity as well as a higher level of confidence with the general function claim “Omega-3 fatty acids help to maintain a healthy cardiovascular system”. This might be due to the fact that Omega 3 is a substance associated with various health benefits [30,44,45] and mostly coupled with a great variety of food products in everyday use (from dairy products to eggs). In addition, extensive marketing promotion and communication efforts have built a healthy reputation of the omega-3 concept in recent years and this might influence the credibility of health messages [46,47].

Our result on general function claims contrasts with previous evidence which showed that consumers perceive disease risk reduction claims as being more appealing than general function claims [12,13], but it is in line with other research findings that consumers usually prefer short claims with general mentions of health effects compared to claims with specific information relating to disease risk reduction or containing warnings [48,49].

As for knowledge, in contrast with our results on familiarity, consumers’ objective knowledge of claims is higher for HCs than for NCs. In particular, although “No sugar added” is the most familiar claim, it is the most difficult to interpret. The same holds for the Omega 3 claim among HCs. With respect to no sugar added claims being difficult to interpret, similar results were found by Patterson et al. [50] showing that consumers could underestimate the actual nutritional value of products that carry sugar claims, while for Omega 3 the difficulty among consumers could be connected to the existence of various authorized claims for this substance present on the market that may create confusion.

What was found with reference to familiarity and knowledge leads to two different considerations. On the one hand, our results are in line with previous research findings, according to which consumers show a positive bias towards NHCs connected to potential halo effects of some claims (consumers tend to rate the product higher on attributes mentioned in the claim) and magic bullet effects that occur when consumers attribute inappropriate health benefits to the product [51].

These potential effects need to be taken into particular consideration by policy makers, in light of the strong influence that advertising campaigns may have on consumers’ perception of the health properties of food products. In this regard, it is important to recall that some food companies have in the past conducted advertising campaigns using deceptive health claims, improperly leading individuals to attribute health properties to these products. Indeed, in Italy several companies have been sanctioned pursuant to Regulation 1924/2006 within the consumer protection activity carried out by the Italian Competition Authority [52,53].

In addition, even if consumers may be familiar with the nutrients mentioned in the claims, they may not understand the role that food products or nutrients play in their diets and overall health [6]. As a consequence, efforts should be made to simplify claims and make them more understandable to consumers, and above all to promote an understanding of the real beneficial effect on health. On the other hand, our results contrast with previous research suggesting that familiarity greatly affects consumer knowledge and understanding of NHCs [6].

The above evidence allows a critical issue to be highlighted: As consumers perceive products labelled with NHCs as healthier, their scant knowledge of the actual meaning of such claims could mislead their purchasing decisions [30]. This issue is also recognized by the EC Regulation on NHCs, which prescribes that claims should be used only if the average consumer can be expected to understand the beneficial effect. However, the level of consumer ability to understand claims may depend on several factors such as the use of scientific terms and the length of the claim [54]. Thus, in accordance with Hung et al. [9], it is important to increase consumer awareness and understanding of the actual meaning of NHCs available on the market, in order to protect the public from being misled while evaluating the healthiness of food. This suggests that policy makers as well as food marketers should also focus on facilitating understanding of NHCs, for example through adapting the wording or length of health claims.

Cluster analysis, in line with previous research [5,8,15,55], shows the existence of three consumer profiles with different levels of attention and use frequencies of NHCs. However, in contrast with Hung and Verbeke [8], our results show that the *non-users* segment represents the smallest cluster, while the largest comprises consumers quite interested in claims, which we called *potential users*.

It is worth pointing out that consumers who show a higher degree of familiarity and use of NHCs (the cluster called *claims users*) also pay more attention to nutritional panels on labels. This is an interesting result given that previous research found that the use of nutrition claims together with a detailed nutrition facts panel increases consumer utility beyond the increase provided by each label in isolation [35]. This suggests that nutritional panels and NHCs tend to reinforce each other and that the use and credibility of claims can be strengthened by educating consumers and promoting a more frequent use of nutritional panels.

Consistent with other studies our results show the key role of motivations in influencing NHC use and familiarity. Indeed, *Claims users* show on average the highest level of general health interest in food choices, while *Non-users* have the lowest. Thus, in accordance with Dean et al. [12] and Hung et al. [7], food products with NHCs are more appealing to consumers who are interested in healthy eating, who are also those that show the greatest need of health-related information. As a consequence, consumer attitudes to using NHCs could be encouraged by stimulating consumer interest in healthy eating.

Finally, with regard to the presence of personal and/or household members’ health problems our results confirm previous research findings [14,20] that this condition could affect interest in and use of NHCs. Those in *Claims users* show the highest incidence of respondents who claim to be in this condition. However, also in the *Non-users* cluster a high incidence of individuals with personal and/or household members’ health problems is revealed. Thus the incidence of personal or family experience with health issues on attention to, and use of, NHCs is controversial. In this regard, in accordance with Verbeke et al. [30], the link between diet and health is complex and consumers may react differently, also with regard to different NHCs. This complexity is confirmed by our results according to which, contrasting with other studies [8,12], the existence of special dietary needs does not differ significantly among clusters.

Another interesting difference that emerges among the clusters concerns the consumers’ perceived ability to process NHCs. The *Non-users* cluster shows the lowest level of ability to process NHCs, which may influence their lower use of NHCs, since previous research shows that usage of claims is strictly connected to ability to process [7].

Furthermore, the degree of nutritional knowledge as well as the degree of objective knowledge of NHCs differs among clusters. This confirms that a higher level of nutritional knowledge may support the use of NHCs [15,35] and suggests that policy makers should focus on enhancing nutritional knowledge in the population by implementing public campaigns that allow consumers to process information contents. However, it has been shown elsewhere that the effects of nutrition knowledge on claims depend on the claim type involved [56].

Overall, in line with previous research our results show that consumers with lower education are less motivated towards NHCs [15,20,36], supporting the idea that better educated consumers are generally more likely to search for nutritional and health information [21] and that education is positively related to knowledge and understanding of nutritional information [57].

Finally, our results confirm that males are less motivated towards NHCs [15,20,36], but contrary to other research there are no significant differences in the identified clusters as regards age or the presence of children [9,15,24]. However, with reference to the presence of children we deliberately excluded claims related to children’s development and health in order to prevent responses being affected by the different household composition of the sample.

This study has some limitations that constitute areas for further research. First of all, the influence of product-specific characteristics on NHC use, familiarity and credibility was not investigated. It would be worth ascertaining the influence of carrier products as well as their sensory profile on the use of NHCs. In addition, we considered only a limited number of specific claims, while it would be useful to consider a wider range of claims present on the national market. Moreover, even if we consider both nutrition and health claims, no interaction effects are investigated in the present analysis. Finally, a further limitation concerns the use of self-reported data that may be susceptible to social desirability bias. In this regard, future research should involve the carrying-out of experimental and observational studies.

## 6. Conclusions

The findings of this study suggest the existence of three segments of consumers in Italy, with different levels of attention and use frequency of NHCs, with specific profiles in terms of motivation, nutritional knowledge, and ability to process and understand NHCs. Hence, first of all, policy interventions as well as communication efforts targeting different consumer segments are required to support the use of NHCs and to avoid misleading interpretation and perception. According to our results it would be useful to adopt appropriate communication strategies to promote consumer awareness of the importance of healthy eating, such as public education campaigns, using both traditional and innovative tools. Specifically, new technologies, such as so-called smart labels or QR codes or other apps for mobiles, can make an important contribution, even if it has been suggested that such new labelling technologies could motivate consumers to access the information if combined with additional interventions [58]. Also, a nudge-based approach could represent a valid method to support consumers in making healthier food choices during their purchases, complementing other tools [9,59].

Furthermore, the results show a low level of objective knowledge of NHCs among Italian consumers. It should therefore be considered a priority for policy makers and marketers to inform consumers better about the actual of NHCs, in the context of a healthy diet, in order to improve their use and effectiveness, avoiding misleading interpretation and perception, such as overestimation of the benefit or attribution of inappropriate health benefits. Overall, but especially to reach the *Non-users* cluster, information should be provided simply and clearly, avoiding the use of complex scientific terminology. Finally, it should be emphasized that among the three clusters identified, that of *potential users* is numerically the largest. The communication and information policies outlined above could therefore have a significant impact on increasing understanding and use of NHCs.

## Figures and Tables

**Table 1:** Sample characteristics.

		Sample	National Population
Gender *	Male	49.8	49.6
	Female	50.2	50.4
Age *	18–24	10.0	10.0
	25–34	16.5	16.1
	35–44	20.3	20.7
	45–54	23.5	23.4
	55–70	29.7	29.5
Current education level **	Post graduate specialization/PhD	5.4	*n.a.*
	Master’s Degree	15.7	19.3
	Bachelor’s Degree	10.4	
	High school diploma	58.0	61
	Other	10.6	*n.a.*
Occupation	Housewife/Househusband	12.7	*n.a.*
	Employee	21.3	*n.a.*
	Self-employed	11	*n.a.*
	Worker	11	*n.a.*
	Retired	14	*n.a.*
	Unemployed	12	*n.a.*
	Other	18	*n.a.*
Area of Residence *	Northwest	26.3	26.2
	Northeast	18.9	19
	Centre	22.5	22
	South	32.3	32.8
Children <12	Yes	28	*n.a.*
	No	72	*n.a.*
Household economic status ***	Below national average	30.9	*n.a.*
	In line with national average	63.3	*n.a.*
	Above national average	5.8	*n.a.*
Special dietary needs	Yes	33	*n.a.*
	No	67	*n.a.*
Personal and/or household members’ health problems ****	High blood pressure	26	*n.a.*
	High cholesterol	27	*n.a.*
	Cardiovascular problems	8.4	*n.a.*
	Osteoporosis	9.2	*n.a.*
	Dental caries	15.7	*n.a.*
	Other	5	*n.a.*
	No problems	43	*n.a.*

* Italian National Institute of Statistics official data on the resident population on 1 January 2017 by age (18–70), gender and geographical area [41]. ** Italian National Institute of Statistics official data for 2017 on the population between 25 and 64 years [42]. *** Average net annual income of Italian families according to Italian National Institute of Statistics official data was about €30,000 for 2016. **** Although the incidence of personal or family pathologies is not representative of the national reality, it is possible to find a correspondence between the main pathologies indicated by our sample and those detected by the 2017 ISTAT survey [43] according to which 40% of Italians suffer from a chronic disease. The most prevalent chronic disease is hypertension while osteoporosis accounts for 7.6%.

**Table 2:** Motivation and ability to process nutrition and health claims (NHCs).

Health Interest in Food Choices (Cronbach’s Alpha = 0.834)	Mean	S.D.
I always follow a healthy and balanced diet	3.2	0.96659
It is important for me that my diet is low in fat	3.3	0.97366
It is important for me that my daily diet contains a lot of vitamins and minerals	3.5	0.95734
I am very particular about the healthiness of food I eat	3.4	0.96194
**Need for health-related information (Cronbach’s alpha = 0.838)**		
It is necessary for me to know the nutrient content of food products	3.5	1.01235
It bothers me if health-related information is not available on food labels	3.3	1.11105
It is important for me to be informed if one or more components of the foods I choose reduces a risk factor in the development of a human disease	3.6	1.03137
**Perceived ability to process health and nutrition claims** **(Cronbach’s alpha = 0.863)**	**Mean**	**S.D.**
Compared to most people, I am quite knowledgeable about health and nutrition claims	3.0	0.85479
Compared to most people, I am more confident in using health and nutrition claims in making food choices	3.1	0.85999
I am confident about my ability to understand health and nutrition claims	3.2	0.86439

**Table 3:** Familiarity and credibility towards specific health and nutrition claims.

	Familiarity	Credibility
**Nutrition Claims**	**Mean (S.D.)**	**Mean (S.D.)**
Low in sodium	3.6 (0.956)	3.2 (0.846)
Reduced kcal	3.6 (0.963)	3 (0.892)
No sugar added	3.8 (0.949)	3.1 (0.961)
**Health Claims**		
Plant sterols have been shown to lower blood cholesterol levels. High cholesterol is a risk factor in the development of coronary heart disease	3.4 (1.096)	3.4 (0.930)
Omega-3 fatty acids help to maintain a healthy cardiovascular system	3.6 (1.091)	3.5 (0.899)
Chewing gum sweetened with 100% xylitol helps neutralize plaque acids. Plaque acids are a risk factor in the development of dental caries	3.1 (1.009)	3 (0.952)

All pairwise comparisons among familiarity and credibility means for nutrition claims (NCs) and health claims (HCs) are statistically significant (according to *t*-test *p*-value < 0.05) except for familiarity between Low in sodium/Omega-3 and Reduced kcal/Omega-3.

**Table 4:** Cluster profiling in terms of motivations, evaluation and knowledge of NHCs.

	Potential Users	Claims Users	Non-Users	Total Sample	*p*-Value *
Attention to nutritional panels	3.29 ^a^	4.08 ^b^	2.12 ^c^	3.33	0.000
Attention to NCs	3.49 ^a^	4.25 ^b^	2.14 ^c^	3.48	0.004
Attention to HCs	2.82 ^a^	4.19 ^b^	1.71 ^c^	3.06	0.000
Buying frequency of NC-labelled products	3.31 ^a^	4.20 ^b^	2.13 ^c^	3.38	0.000
Buying frequency of HC-labelled products	2.72 ^a^	4.02 ^b^	1.75 ^c^	2.97	0.000
General health interest **	3.26 ^a^	3.84 ^b^	2.70 ^c^	3.34	0.000
Need for health-related information **	3.45 ^a^	4.11 ^b^	2.70 ^c^	3.52	0.000
Perceived ability to process health and nutrition claims **	3.10 ^a^	3.60 ^b^	2.53 ^c^	3.16	0.000
Familiarity with specific NCs ***	3.55 ^a^	4.06 ^b^	3.12 ^c^	3.64	0.000
Credibility of specific NCs ***	3.04 ^a^	3.51 ^b^	2.69 ^c^	3.13	0.003
Familiarity with specific HCs ***	3.30 ^a^	3.74 ^b^	2.92 ^c^	3.37	0.000
Credibility of specific HCs ***	3.18 ^a^	3.56 ^a^	2.86 ^b^	3.24	0.000
Nutritional Knowledge Index	2.38 ^a^	2.54 ^a^	2.04 ^b^	2.37	0.004
NHC Knowledge Index	2.75 ^a^	2.76 ^a^	2.32 ^b^	2.67	0.013
NC Knowledge Index	1.07	1.04	0.90	1.03	0.177
HC Knowledge Index	1.23 ^a^	1.28 ^a^	1.05 ^b^	1.21	0.085

*p*-value are related to F test in one-way ANOVA. ** Based on the mean value of items used; *** Based on the mean value of familiarity and credibility of each nutrition (low in sodium, reduced kcal content and no sugar added) and health claims (related to plant sterols; omega-3 fatty acids and xylitol). Different subscripts indicate a significant difference at *p* < 0.05 using Tukey’s HSD test.

**Table 5:** Cluster profiles based on socio-demographics.

		Potential Users	Claims Users	Non-Users	*p*-Value
Gender *	Male	47	46	61	0.032
	Female	53	54	39	
Mean age		44.76	46.20	45.45	0.606
Education Level *	Post-graduate specialization/PhD	5.1	8.3	1.0	0.044
	Master’s degree	16.5	15.5	14.3	
	Bachelor’s degree	14.0	7.1	7.1	
	High school diploma	55.1	58.9	63.3	
	Other	9.3	10.1	14.3	
Children <12	Yes	30	27	23	0.436
	No	70	73	77	
Special dietary needs	Yes	15	19	13	0.075
	No	85	81	87	
Personal and/or household members’ health problems *	Yes	54	60	57	0.046
	No	46	40	43	

* *p*-value < 0.05 for chi-square test.
